# Comparison of the Variability of Small Extracellular Vesicles Derived from Human Liver Cancer Tissues and Cultured from the Tissue Explants Based on a Simple Enrichment Method

**DOI:** 10.1007/s12015-021-10264-1

**Published:** 2021-09-22

**Authors:** Jie Chen, Zhigang Jiao, Jianwen Mo, Defa Huang, Zhengzhe Li, Wenjuan Zhang, Tong Yang, Minghong Zhao, Fangfang Xie, Die Hu, Xiaoxing Wang, Xiaomei Yi, Yu Jiang, Tianyu Zhong

**Affiliations:** 1 The First School of Clinical Medicine, Gannan Medical University, Ganzhou, China; 2 Laboratory Medicine, First Affiliated Hospital of Gannan Medical University, Ganzhou, China; 3 Precision Medicine Center, First Affiliated Hospital of Gannan Medical University, Ganzhou, China; 4 Department of Orthopedic Surgery, First Affiliated Hospital of Gannan Medical University, Ganzhou, China; 5 Department of Pharmacology and Chemical Biology, University of Pittsburgh School of Medicine, Pittsburgh, PA USA

**Keywords:** Small extracellular vesicles, NanoFCM, Isolation, Purification, Liver cancer tissue

## Abstract

A potential use of small extracellular vesicles (sEVs) for diagnostic and therapeutic purposes has recently generated a great interest. sEVs, when purified directly from various tissues with proper procedures, can reflect the physiological and pathological state of the organism. However, the quality of sEV is affected by many factors during isolation, including separation of sEV from cell and tissues debris, the use of enzymes for tissue digestion, and the storage state of tissues. In the present study, we established an assay for the isolation and purification of liver cancer tissues-derived sEVs (tdsEVs) and cultured explants-derived sEVs (cedsEVs) by comparing the quality of sEVs derived from different concentration of digestion enzyme and incubation time. The nano-flow cytometry (NanoFCM) showed that the isolated tdsEVs by our method are purer than those obtained from differential ultracentrifugation. Our study thus establishes a simple and effective approach for isolation of high-quality sEVs that can be used for analysis of their constituents.

**Graphical abstract:**

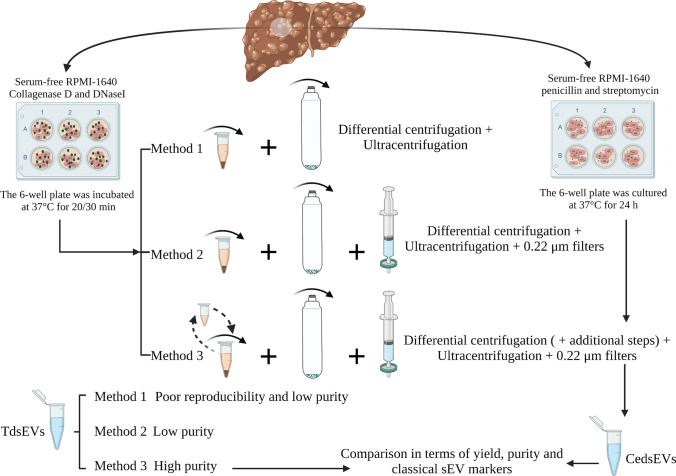

**Supplementary Information:**

The online version contains supplementary material available at 10.1007/s12015-021-10264-1.

## Introduction

Small extracellular vesicles (sEVs) are a kind of bilayer phospholipid membrane vesicles (30–200 nm in diameter) [1, 2] that are derived from various types of cells and released into the various body fluids and the interstitial space of tissues [3–6]. The released sEV play an important role in intercellular communication [7, 8]. These vesicles are packaged with proteins, mRNA, miRNA, rRNA and DNA specific for the cells, which partially reflect the physiological and pathological state of the body [9–11]. Therefore, the nucleic acid and protein constituents of sEVs are considered to have valuable properties for the diagnosis and treatment of disease. The isolation of sEVs from specific cells or tissues represents a big challenge. However, due to the physical characteristic of sEV such as smaller size and low buoyant density, the isolation of sEVs from mixed components in the body fluids is challenging and currently requires tremendous effort. Recently, variety of methods have been developed to separate sEVs from body fluids. Depending on the mechanisms, current sample preparation kits and procedures for sEV isolation can be divided into physical and affinity separation approaches. The physical isolation methods include differential ultracentrifugation (DUC), ultrafiltration (UF), polymer-based precipitation, size exclusion chromatography (SEC) and alternating current electrokinetic (ACE) microarray chip or in combination of them. However, non-sEV protein contamination and the recovery ratio of sEV in sample preparation with these methods still need to be improved. The affinity capture separation approach utilizes nanoparticle coated with antibodies against the surface proteins of sEVs [12, 13]. However, the approach may miss some potentially important subpopulation of sEVs. In addition, the selectivity, specificity, and the affinity/binding constant of coated-antibody remain to be confirmed.

The sEV pool in the body fluids is a mixture which contains various sEV derived from different cells and tissues. This large heterogeneity in the sEV pool represents a challenge for isolating and identifying a subtype of sEV that may have a clinical value. Because tissue-derived sEVs are presented in the interstitial space and are most likely to act locally, the composition of these sEVs is considered to reflect physiological and pathological activities in the organ from which the sEVs are derived. Moreover, sEVs in tissue could be used to identify reliable cell-specific markers that could then be used to capture specific populations of tissue-origin sEVs in the periphery, which can be used to diagnose and monitor disease conditions [14–16]. Nevertheless, the separation efficiency of sEV from tissue may be influenced by the state of tissue specimen such as fresh, frozen or slice cultivation samples derived from fresh biopsy tissue. Moreover, the concentrations of collagenase D and DNase I and incubation time with samples may affect the quality of isolated sEVs. Collagenase D and DNase I are commonly used to isolate cells from tissue. Collagenase D digests the intercellular matrix and degrades natural collagen with little damage to cells, while DNase I is able to reduce the viscosity of the tissue suspension, hence facilitating isolation of cells [17]. Currently, they are also used to extract sEVs from human metastatic melanoma tissues [18, 19], colon cancer tissues and colonic mucosa tissues [17]. A combined use of these enzymes results in releasing more tdsEVs, which has been used for isolation of sEVs in some studies. However, the concentrations of collagenase D and DNase I and digestion times vary in these approaches, which lead to inconsistent conclusions from these studies.

It is worth noting that the purity and cell specificity of EV preparations are vital for subsequent proteomic, genomic and lipidomic analyses. In this study, we designed a simple method for gaining high-purity sEV from liver cancer tissues. We used the ratio of CD9/CD63 positive sEVs by NanoFCM to confirm the purity of sEVs isolated through combination of different concentrations of enzymes and incubated times. The protocol that gains high-purity sEV was used to isolate sEVs from liver cancer tissues for characterization.

## Materials and Methods

### Tissue Collection and Preparation

Human liver cancer tissues were collected from patients diagnosed with liver malignancy at the First Affiliated Hospital of Gannan Medical University. Informed written consents were obtained from the patients and the collection of human tissue samples was approved by the Ethics Committee of the First Affiliated Hospital of Gannan Medical University. All samples were transported in pre-cooled PBS. The samples were divided into three parts, which were used for the preparation of fresh tdsEVs (tissue samples collected within 2 h), frozen tdsEVs (tissue samples stored at −80 °C for at least 24 h) and cedsEVs (tissue samples collected within 2 h).

### Cell Culture

Human umbilical vein endothelial cells (HUVECs) were cultured in Dulbecco’s Modified Eagle Medium (DMEM; Gibco, USA) containing 10% fetal bovine serum (FBS; Excell Bio, Uruguay), 100 units/mL penicillin, and 100 μg/mL streptomycin. The cells were maintained in cell culture dishes (BIOFIL, China) in a humidified chamber at 37 °C with 5% CO_2_.

### Cell Migration Assay

Cell migration was identified by a wound-healing assay. HUVECs (4 × 10^5^ cells per well) were grown to 90% confluence in a 6-well plate. Wounds were made on cell monolayers by topping a 200 μL pipette. The Cells were cultured with DMEM. Wound images were captured by microscopy (NOVEL, China) at 0 h, 24 h and 48 h after wounding. The wound area was measured using the ImageJ software, and the percentage of wound closure was calculated using the following formula: Area recovery (%) = 100% − (wound area after 24 h or 48 h/wound area at the beginning) × 100%.

### PKH67-Labeled sEVs

The isolated tdsEVs were labeled with a PKH67 green fluorescent labeling kit (Sigma-Aldrich) following the instructions. In brief, 100 μL of tdsEVs and 4 μL of PKH67 were diluted in 1 mL of Dilution C, respectively, and then they were mixed evenly. Their mixture was incubated for 4 min at room temperature. Whereafter, 2 mL of 1% bovine serum albumin (BSA) was added to bind the excess PKH67. The sample was centrifuged twice at 100,000×g, 70 min to remove excess PKH67. PKH67-labeled sEVs were resuspended in DMEM medium and added to HUVECs (cultured in a 6-well plate). After co-incubation for 6 h, the cells were fixed with 2% paraformaldehyde for 10 min and then blocked with mounting medium (with DAPI). Images were taken with confocal fluorescence microscope.

### Separation and Purification of tdsEVs and cedsEVs

**Fig. 1:**
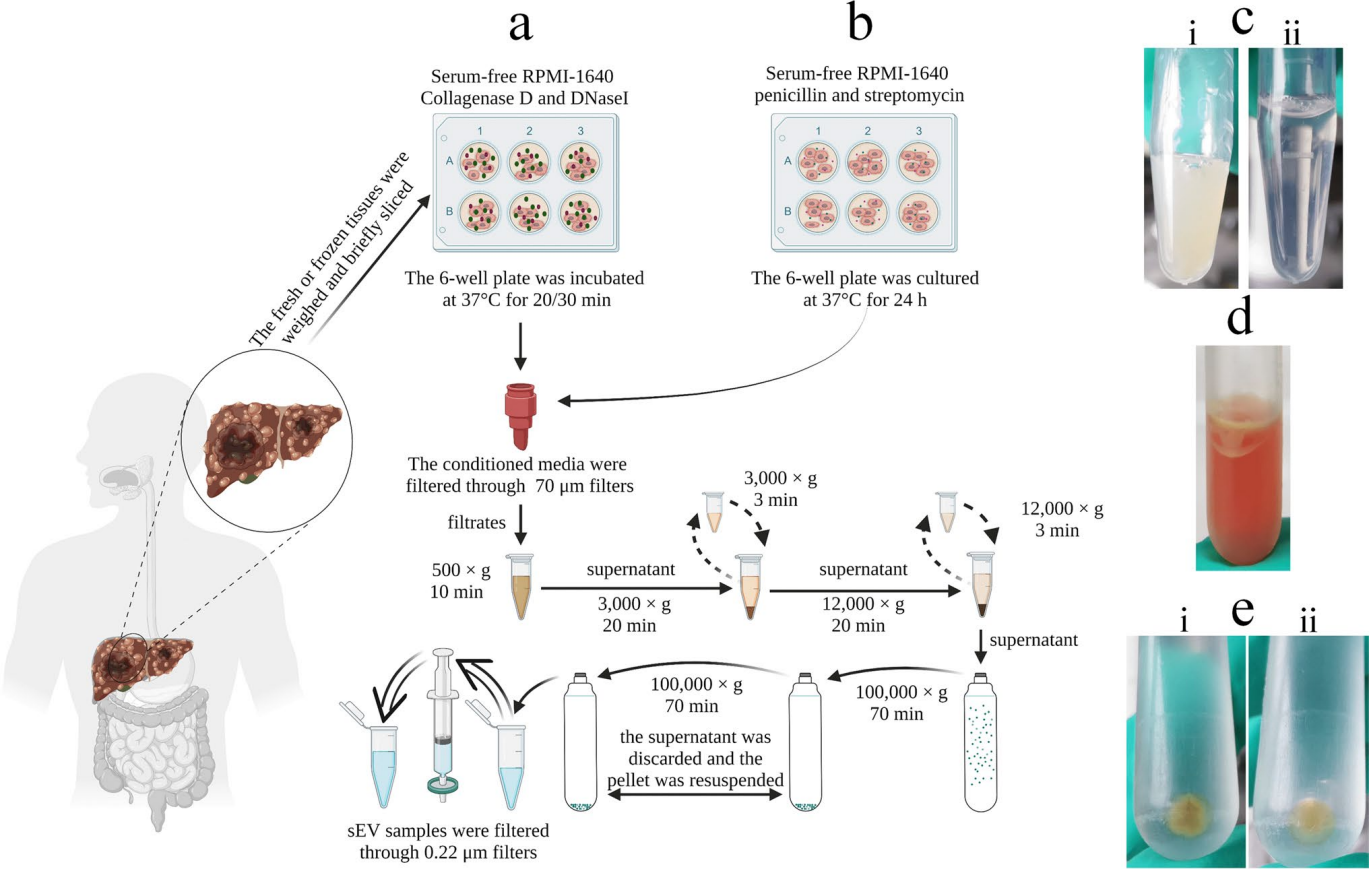
Schematic overview of enrichment methods for tdsEVs and cedsEVs. (**a**) Three different methods for the isolation and purification of tdsEVs. Method 1 is a classical differential ultracentrifugation method, i.e., a single solid arrow process; Method 1 was added a 0.22 μm filtration step (double solid arrow) to be method 2; Method 3 combines method 2 with the two-step differential centrifugation step (single dashed arrow). (**b**) Method used for preparation of cedsEVs. Differential ultracentrifugation and filtration steps are the same as method 3. (a + b) The samples were taken from 3 different patients with liver cancer and the same patient samples were used for the 3 methods in each assay. (**c**) Comparison of before (i) and after (ii) filtration of tdsEVs through 0.22-μm filters. (**d**) White contaminants in the supernatant. (e) The pellets produced by differential ultracentrifugation steps of method 1 (i) and method 3 (ii)

To obtain tdsEVs with high purity, three different methods were used (Fig. 1a). Briefly, the tissues were weighed and cut into small pieces (2 × 2 × 2 mm) on ice, then placed in RPMI-1640 medium containing collagenase D (1 mg/mL, 2 mg/mL and 4 mg/mL, respectively) and DNase I (20 U/mL, 40 U/mL and 80 U/mL, respectively) and incubated at 37 °C for 20 min or (and) 30 min (only one of the combinations was used after the experimental conditions were established). The samples were put on ice immediately after the incubation. PhosSTOP and Complete Protease Inhibitor (Sigma-Aldrich PS/PI 4906837001/11697498001) solution were then added to stop the digestion. The digestion solution was filtered through a 70 μm cell strainer (Biosharp, BS-70-XBS) to remove larger tissue debris. The filtrate was processed by differential centrifugation (500×g, 10 min; 3000×g, 20 min; 12,000×g, 20 min) to remove cellular debris, large vesicles, and some impurity particles. The obtained supernatant was centrifuged twice at 100,000×g for 70 min, and the precipitation was resuspended with PBS. Temperature for all centrifugations was 4 °C. The above procedure was referred as method 1. For the second method (method 2), the samples were filtered with 0.22-μm filters (Millipore, SLGPR33RB) to remove impurity particles and insoluble matter (Fig. 1c). Besides the 0.22-μm filtration process, the third method (method 3) also added a series of additional centrifugation steps to further remove some contaminations. During this process, a portion of the supernatant after completion of centrifugation (3000×g, 12,000×g) was taken and centrifuged again at the same centrifugal force for 3 min. When aspirating the liquid, as little of the upper white layer as possible was recovered so as not to affect purity of sEV (Fig. 1d). The precipitates produced by the differential ultracentrifugation steps of method 1 (i) and method 3 (ii) were shown in Fig. 1e. The whole separation scheme for tdsEVs (Fig. 1a) and cedsEVs (Fig. 1b) was modified from the protocol established by Vella et al. [6] and Hoshino et al. [15].

### NanoFCM Analysis

NanoFCM with high throughput, high sensitivity and high resolution was used for the analysis of EVs [20, 21]. The number of each sEV sample was determined by calibrating the sample flow rate using a green fluorescent sphere with a known particle concentration (1.9 × 10^10^ particles/mL). Size distribution was calculated by standard curves generated using several nanomicrospheres of different diameters (68–155 nm) under the same detection condition. Distribution histograms or dot-plots were derived from data collected at 1 min for all samples. For immunofluorescent staining, antibodies were purchased from BD Pharmingen™: FITC Mouse Anti-Human CD9 (Clone M-L13), FITC Mouse Anti-Human CD63 (Clone H5C6). Add 20 μL of FITC-labeled anti-CD9 and CD63 antibodies to 5 μL of different sEV samples with particle concentration of approximately 1 × 10^10^ particles/mL. These mixtures were incubated at 37 °C for 30 min. Care was taken to avoid light throughout. The samples were washed twice with PBS at 100,000×g for 30 min at 4 °C (Beckman Coulter MAX-TL centrifuge, TLA-110 rotor). The pellet was resuspended in 100 μL PBS for NanoFCM (N30E, NanoFCM Inc., China) analysis. 1% Triton X-100 has been previously used for the assessment of EVs purity [20, 22]. Treatment of samples with the detergent resulted in rupture of the sEVs, while non-membranous contaminants remained intact [23]. The purity of sEVs was calculated as (1-C2/C1) × 100%, where C1 and C2 represent the particle number detected in 1 min before and after Triton X-100 treatment, respectively. In detail, 5 μL of 10% Triton X-100 was added to 45 μL of diluted sEVs, while a mixture of 5 μL of PBS and 45 μL of the sample was incubated on ice for 1 h as a control. The treated sEV samples were diluted 100-fold and used for NanoFCM assay.

### Transmission Electron Microscopy (TEM)

10 μL of sEVs sample was placed on formvar−/carbon-coated grid, which was cleaned in advance with a plasma surface treatment instrument (PDC-32G-2, Harrick Plasma, USA). The sample was allowed to settle for 10 min before stained with 2% phosphotungstic acid for 1 min. Grids were imaged with a JEM 1200EX (Jeol Ltd., Japanese) transmission electron microscope operating at 120 kV.

### Western Blot Analysis

The protein concentrations of sEV samples and cells were measured with Pierce™ BCA Protein Assay Kit (Product No. 23,227, Thermo Scientific, USA). Protein standard samples ranging from 0.0625 mg/mL to 1 mg/mL were used. The protein of each sample was adjusted to 5 μg loaded onto a 12% polyacrylamide gel for electrophoresis. Proteins were then transferred from the gel to a PVDF membrane (MILLIPORE) using a constant current of 200 mA under low temperature. The membrane was blocked with 5% non-fat dry milk in TBST for 1 h at room temperature and incubated with primary antibody overnight at 4 °C. They were washed with 1 × TBST, and incubated with horseradish peroxidase (HRP)-conjugated secondary antibody at room temperature for 1 h. The HRP-linked antibody was detected by incubation with New Cell & Molecular Biotech’s chemiluminescent substrate and the images were taken with JP-K300 (Shanghai JiaPeng Science technology). The following antibodies used for immunoblotting were purchased from Abcam. Rabbit monoclonal anti-human CD9 antibody (Clone EPR2949, dilution 1:1000), rabbit monoclonal anti-human CD63 antibody (Clone EPR5702, dilution 1:1000), rabbit monoclonal anti-human TSG101 antibody (Clone EPR7130(B), dilution 1:1000), rabbit monoclonal anti-human HSP70 antibody (Clone EPR2914, dilution 1:1000). The following antibodies were purchased from Proteintech. Mouse monoclonal anti-human GM130 antibody (Clone 2A4F11, dilution 1:1000) and the secondary antibodies: horseradish peroxidase-labeled goat anti-rabbit and goat anti-mouse.

### Statistical Analysis

Data were analyzed with GraphPad Prism version 8.0 (GraphPad Software). ANOVA and unpaired two-tailed Student’s t test were applied to test differences in sEV samples. Differences with *P* < 0.05 were considered statistically significant. GraphPad Software was used for calculation of the mean and the standard error of mean (SEM). Figures were prepared using GraphPad Software, Photoshop and Flow Jo.

## Results

### Establishment of Isolation and Purification Methods

**Fig. 2:**
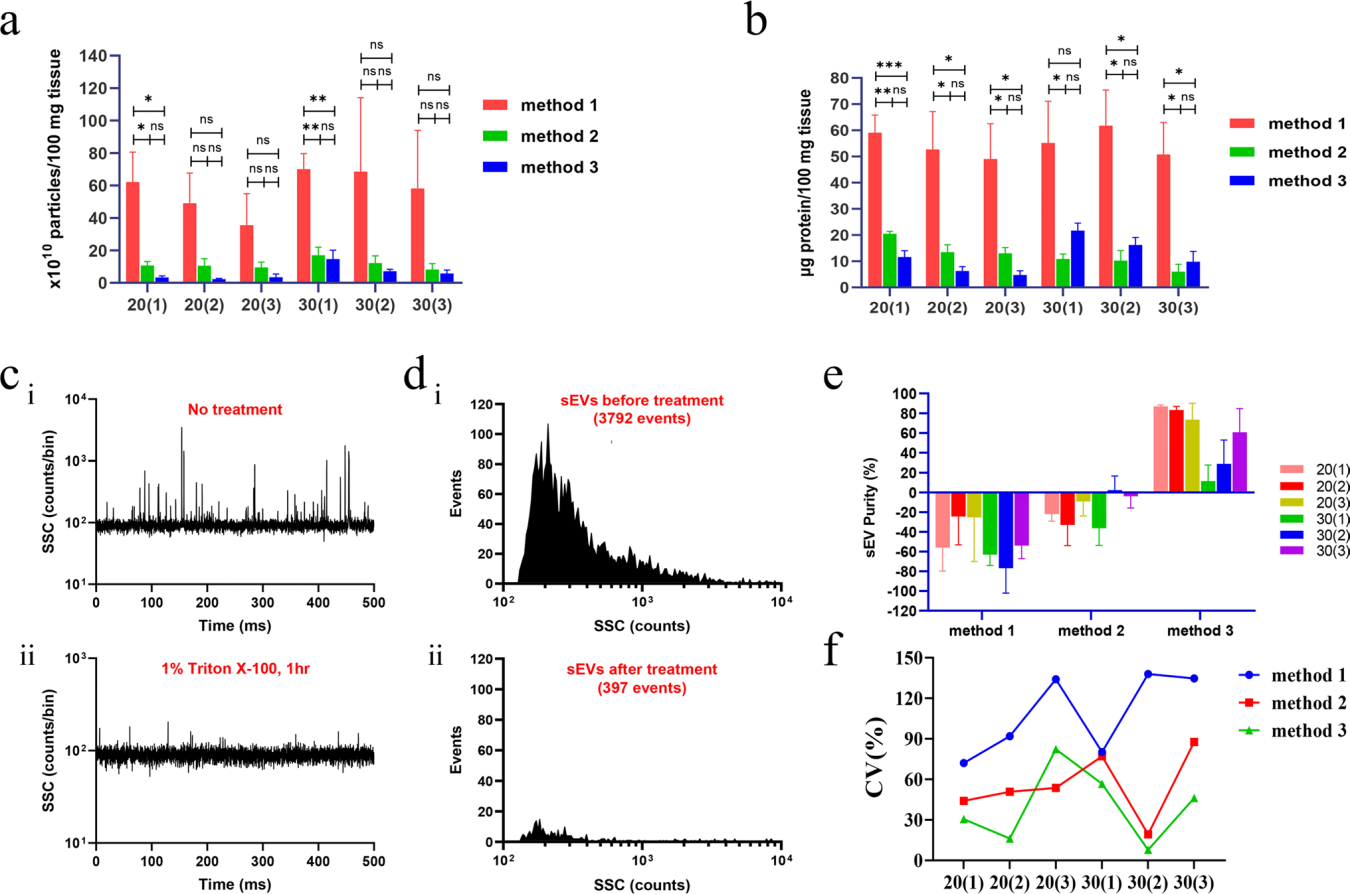
Particle concentration, protein content and purity analysis of the three different methods for isolation of tdsEVs. (**a**) Particle concentration of tdsEVs isolated by three methods at different combinations of enzyme concentrations and incubation times were measured by NanoFCM. The results for each group were normalized by tissue weight (per 100 mg). (1), (2) and (3) represent the concentration combinations of collagenase D (4 mg/mL, 2 mg/mL and 1 mg/mL) and DNase I (80 U/mL, 40 U/mL and 20 U/mL). 20/30 is the incubation time of 20/30 min. For instance, 20(1) is collagenase D (4 mg/mL), DNase I (80 U/mL) and incubation time (20 min). (**b**) Protein content of tdsEVs derived from different methods in the different combinations was determined by the BCA protein assay (per 100 mg tissue). (a + b) data are shown as mean ± SEM. *p < 0.05, **p < 0.01, ***p < 0.001 by unpaired two-tailed Student’s t test. (**c**) Representative SSC burst traces of tdsEVs preparation by method 3 before (i) and after (ii) 1% Triton X-100 treatment for 1 h on ice. (**d**) SSC distribution histograms of tdsEVs preparation by method 3 before (i) and after (ii) Triton X-100 treatment. (**e**) Purity measurement by 1% Triton X-100 for tdsEVs in the different combinations (n = 3, mean ± SEM). (**f**) The coefficient of variation (CV) % distribution of ratio of particle: protein for tdsEVs derived from different methods in the different combinations (n = 3)

To obtain high purity of sEV, we designed and compared three methods for isolation sEVs from tissues treated with different concentrations of enzymes and incubation times. The particle numbers and total protein content are commonly used to evaluate the quantity of isolated sEVs. Therefore, we firstly measured the numbers of particles and protein content by NanoFCM and BCA methods. In these experiments, NanoFCM and BCA methods showed that not only were the numbers of particle and concentration of protein isolated by method 1 significantly higher compared with the other two methods (Fig. 2a-b), but were the CV% values of method 1 derived from the ratio of particles larger (Fig. 2f). Thereafter, the isolated sEVs were treated with 1% of Triton-100 and examined by NanoFCM for evaluating the ratio of vesicles in the total particles. Figure 2c-e and Fig. S1 showed that the ratio of vesicles in the total particles obtained by method 3 was the highest. These results indicate that the sEVs obtained through method 3 have a higher quality. Based on these results, the method 3 was chosen to perform for the subsequent isolation of sEVs.

### Determination of Incubation Concentration and Time for Collagenase D and DNase I

**Fig. 3:**
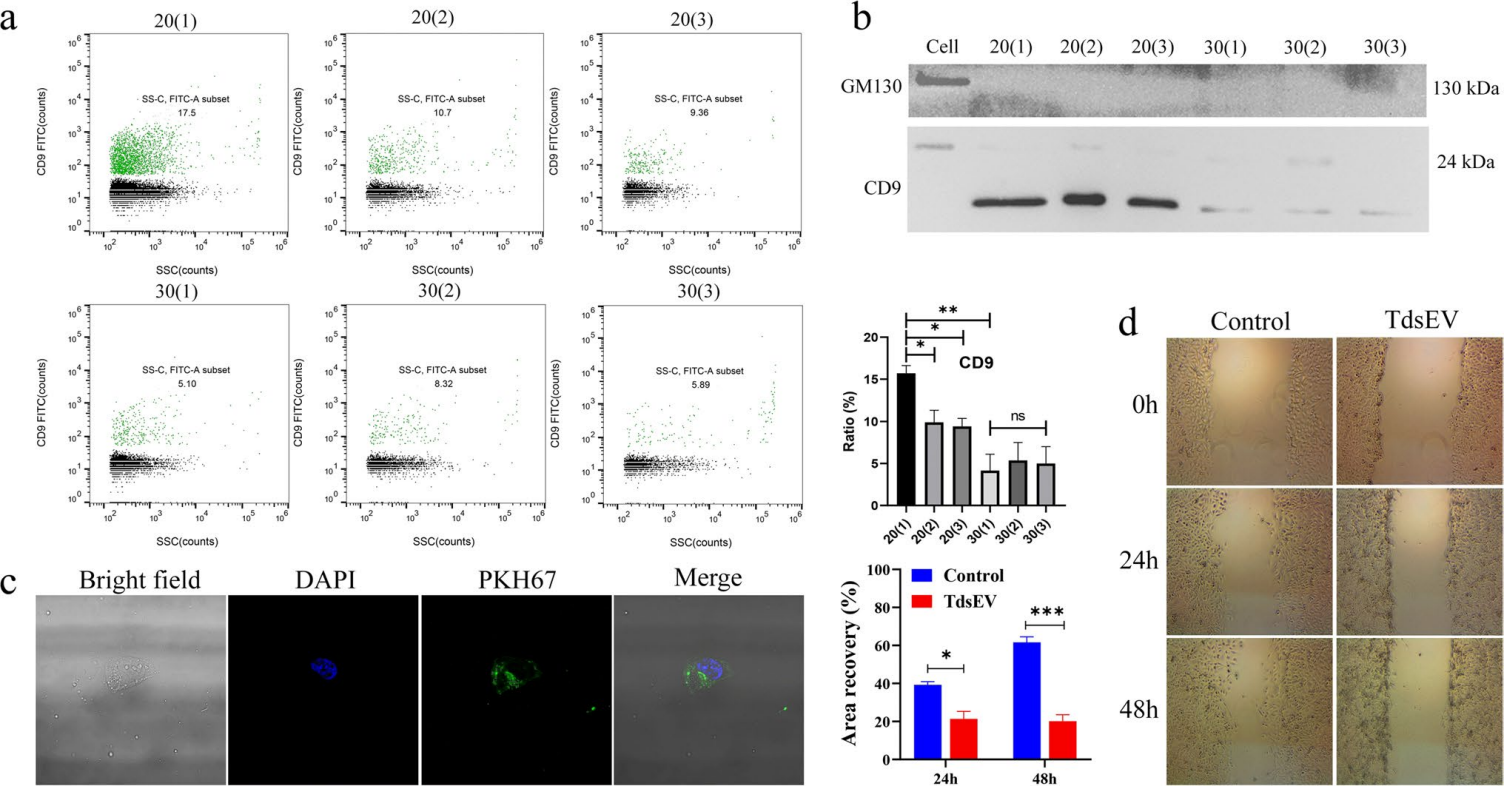
Investigation of conditions for isolation of tdsEVs by NanoFCM. (**a**) Bivariate dot-plots of FITC fluorescence versus SSC for tdsEVs preparation by method 3 (Left panel). The sEVs were labeled with FITC-conjugated mAbs specific to CD9. The percentages of phenotype-positive sEVs are provided in each plot; Measured percentages of a specific phenotype-positive sEVs (n = 3) for CD9 of the different combinations were analyzed by unpaired two-tailed Student’s t test and expressed as mean ± SEM. *p < 0.05, **p < 0.01 and ns: no significant difference (Right panel). (**b**) Western blot assay of CD9 and GM130 associated with cell and sEVs from the different combinations (5 μg protein was loaded per lane). (**c**) Liver cancer tdsEVs isolated by method 3 were labeled with the dye PKH67. These vesicles were then added to HUVECs and incubated for at least 6 h. The samples were analyzed by immunofluorescence under confocal microscopy. (**d**) The wound closure assay was performed to detect cell migration (Right panel). After treatment with tdsEVs and control (as blank control), the area of wound was measured by ImageJ. The area recovery was calculated in the left panel. *p < 0.05, ***p < 0.001 (n = 3, mean ± SEM)

To investigate the efficiency on the purification of tdsEVs from tissues treated by different concentrations of enzymes and incubation times, sEVs were isolated from liver cancer tissues treated with combination of different concentrations of enzymes and incubation times, and analyzed for the biomarkers of sEV such as CD9 and CD63 by NanoFCM. CD81 was not used as a reference because of the interference of digestive enzymes [17]. Representative images of burst traces of tdsEVs and PBS by NanoFCM were depicted in Fig. S2a-b. NanoFCM revealed that the proportions of CD9^+^ vesicles in total sample prepared from six groups were 15.7%, 9.9%, 9.43%, 4.16%, 5.38%, and 5.01%, respectively (Fig. 3a). PBS as a blank control was shown in Fig. S2c. The ratio of CD9^+^ vesicles in 20(1) group was the highest and significantly different from other groups (Fig. 3a). Interestingly, the proportions of CD63^+^ vesicles in all group samples were no difference (Fig. S2c). Western blot analysis showed that the content of the CD9 protein in 20(1) group was also the highest (Fig. 3b). These results indicate that the treatment conditions of 20(1) are more suitable for the isolation of sEVs derived from liver cancer tissues. Next, we used the method 3 isolation approach together with the treatment conditions of 20(1) to isolate tdsEVs. To learn whether these vesicles were functionally active, we co-incubated PKH67-labeled tdsEVs with HUVECs and found that these vesicles were incorporated into the cell (Fig. 3c). Furthermore, wound-healing assay showed that the wound closure was slower in HUVECs treated with tdsEVs compared with the HUVECs’ self-migration (Fig. 3d). The inhibition effect of the sEVs became more pronounced with increasing treatment times (Fig. 3d). These data suggest that the tdsEVs obtained by method 3 are functionally active.

### Particle Concentration, Size, Protein Content and Purity Analysis of sEVs Prepared from Different Tissue States

**Fig. 4:**
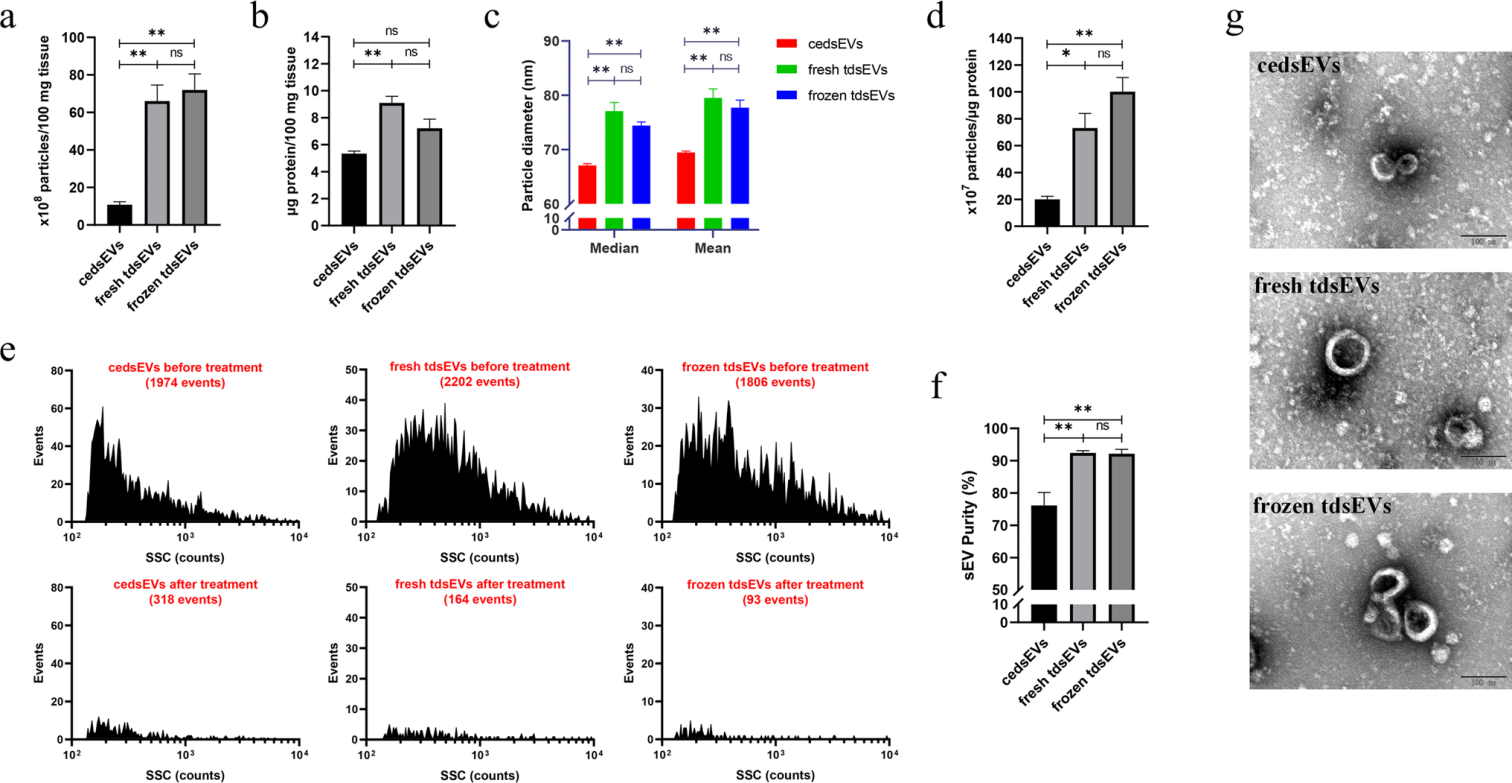
Particle number, protein content, diameter distribution and purity of cedsEVs, fresh and frozen tdsEVs. (**a**) The particle number of isolated fractions was measured by NanoFCM. Particle concentration in each group were normalized by tissue weight (per 100 mg). (**b**) The total protein content of isolated particles in three groups was measured by BCA assay (per 100 mg tissue). (**c**) Median and mean values of particle diameter distribution for three groups. (**d**) The particle number/μg protein ratio for three groups. (**e**) Representative SSC distribution histograms of the sEVs before and after Triton X-100 treatment derived from data collected over 1 min each. The particle number is shown in each plot. (**f**) Purity measurement for three groups by 1% Triton X-100. (**a**-**f**) n = 3, *p < 0.05, **p < 0.01 by ANOVA test. (**g**) CedsEVs, fresh and frozen tdsEVs were visualized by negative staining transmission electron microscopy (scale bar = 100 nm) which is representative of five images taken of each fraction

In order to confirm the most suitable state of tissues for isolation of sEV, we separated sEVs by method 3 isolation protocol and the tissue treatment conditions of 20(1) from three states of liver cancer tissues, including fresh tissues, frozen tissues and cultured tissue slices. The prepared sEVs were analyzed by NanoFCM, BCA assay and TEM. NanoFCM showed that the particle concentration of cedsEVs was significantly lower than those of fresh (*p* = 0.0037) and frozen tdsEVs (*p* = 0.0022) (Fig. 4a). The latter two had a similar particle concentration (Fig. 4a). The BCA results showed that protein content of cedsEVs was significantly lower than that of fresh tdsEVs (*p* = 0.0041), while those of cedsEVs and fresh tdsEVs, compared to frozen tdsEVs, were not significantly different (Fig. 4b). When the sizes of these sEVs were compared, it was found that the mean and median size values of fresh and frozen tdsEVs were significantly greater than that of cedsEVs (the median values are 77.08, 74.42, 67.08, respectively; the mean values are 79.52, 77.73, 69.45, respectively) (Fig. 4c). Next, we assessed the quality of vesicles by means of the ratio of the vesicles in total particles based on the solubility of the particles in 1% of Triton X-100 and the ratio of particle: protein that is a commonly used measure for preparation purity of EVs [24, 25]. Our data showed that the ratios of detergent soluble vesicles in the total numbers of particles of fresh and frozen tdsEVs were significantly higher than that of cedsEVs, while the ratios of those from fresh and frozen tdsEVs were not significantly different (Fig. 4e-f). The result is consistent with measured purity by the ratio of particle: protein (Fig. 4d). TEM also revealed that cedsEVs had smaller particle size than tdsEVs (Fig. 4g), which was consistent with the NanoFCM analysis. These results show that fresh tdsEVs are significantly better than cedsEVs in terms of particle number and protein content. Meanwhile, tdsEVs have a better size distribution and purity. However, fresh and frozen tdsEVs are not significantly different in terms of the foregoing four factors.

### Comparison of Protein Markers for Three Types of sEV

**Fig. 5:**
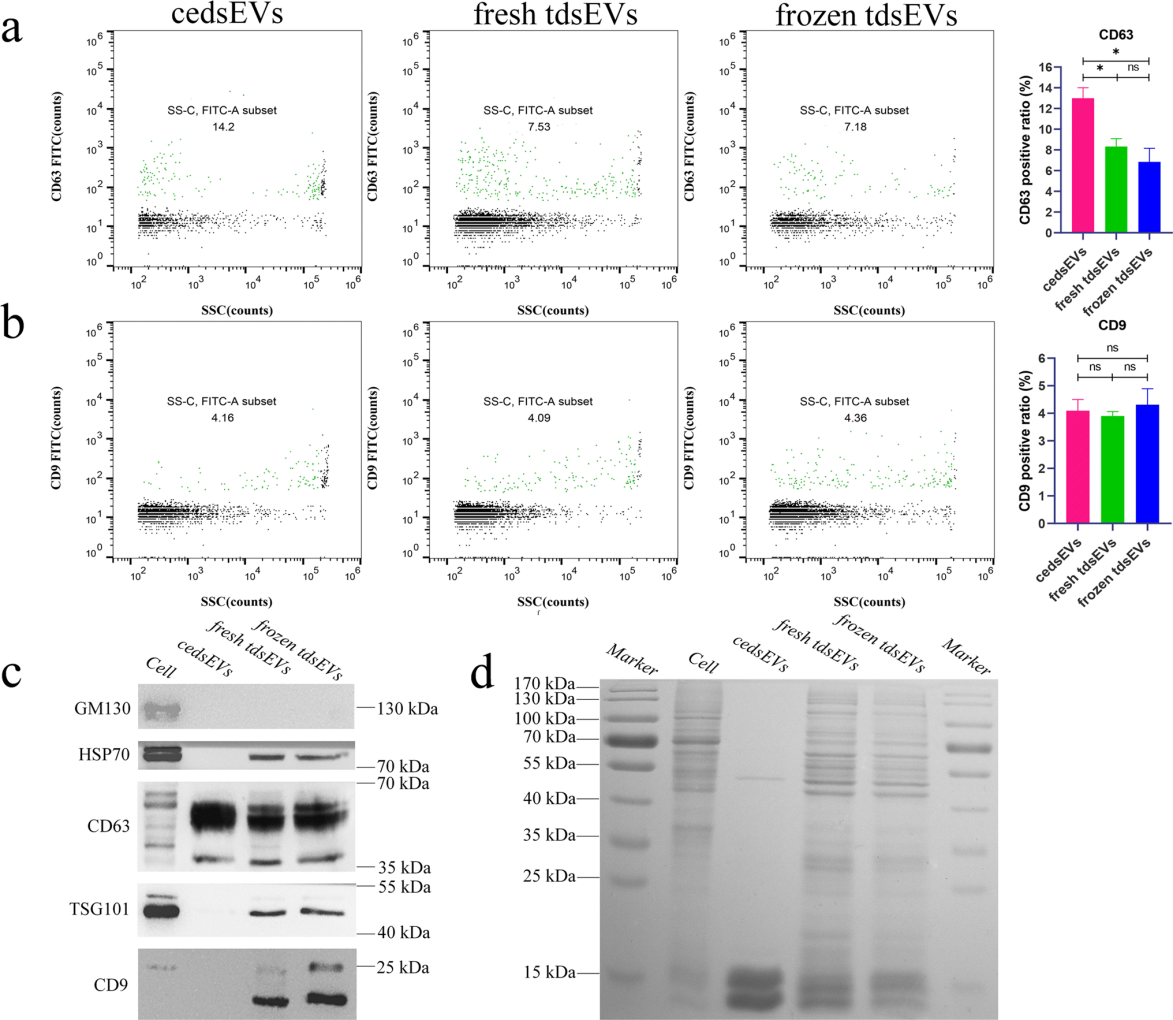
Comparison of sEV marker proteins of cedsEVs, fresh and frozen tdsEVs. (a + b) sEVs were labeled with FITC-conjugated mAbs specific to CD63/CD9 and analyzed by bivariate dot-plots of FITC fluorescence versus SSC. The percentages of CD63 (**a**) and CD9 (**b**) positive sEVs are shown in each plot (Left panel); Percentages of specific fluorescence-positive sEVs (n = 3) for CD9 and CD63 of three types of sEVs (mean ± SEM). *p < 0.05 by ANOVA test (Right panel). (**c**) Western blot analysis of GM130, HSP70, CD63, TSG101 and CD9 in cell extracts and the three types of sEVs. (**d**) Coomassie blue staining of the total proteins from cell extracts and three types of sEVs. (c + d) 5 μg protein was loaded per lane

In the next experiments, we assessed the protein biomarker of sEV with FITC-conjugated mAbs specific to CD63 and CD9 derived from fresh liver cancer tissue, frozen liver cancer tissue and cultured liver cancer tissue sections. NanoFCM revealed that the fluorescence intensities of CD63 of three groups were 13%, 8.32%, and 6.84%, respectively, and the fluorescence intensity of CD63 of cedsEVs was significantly higher than other groups (Fig. 5a). However, the fluorescence intensities of CD9 of three groups were no significant difference from each other (Fig. 5b). Meanwhile, PBS as a blank control was shown in Fig. S3.

The protein markers of EVs such as HSP70, CD63, TSG101 and CD9 were detected by Western blot. GM130 was utilized as a negative control marker for sEV [26], which was present in the cell extracts but absent in sEVs. Western blot showed that protein content of CD9, TSG101 and HSP70 in fresh and frozen tdsEVs were significantly higher than that in cedsEVs and that of CD63 is higher in cedsEVs (Fig. 5c). Coomassie blue staining indicated that fresh and frozen tdsEVs contained more protein species than those of the cedsEVs (Fig. 5d).

## Discussion

Currently, there is a tremendous interest in developing biomarkers from sEVs for diagnosis of disease conditions. The quality of isolated sEVs is vital in these processes. The quality of sEVs can be affected by isolation methods, the concentrations of enzymes and incubated times for digestion of tissues, and storage state of tissue. These factors may lead to an inconsistent quality of sEVs and consequently affect their characterization. In general, the methods for enriching EVs from tissues involve density gradient ultracentrifugation (DGU) [19, 27], commercial sample preparation kits and other recovery methods. Although the purity of EVs obtained with DGU methods is high, the purification process is time-consuming and requires complex experimental procedures and techniques, which make a challenge to transform towards clinical applications. The commercial preparation kits from different manufacturers and batch numbers vary in quality, causing variations of harvested EVs [22, 28, 29]. In the present study, we developed a simple method (method 3) for isolation of sEVs with high purity using human liver cancer tissues. The particle concentration, protein content, size distribution and purity of tdsEVs gained by this method were (3.28 ± 0.84) × 10^10^ particles/100 mg tissue, 11.62 ± 1.94 μg protein/100 mg tissue, 73.05 ± 2.01 nm (mean), 69.08 ± 1.53 nm (median) and 86.89 ± 1.17%, respectively. In comparison with sucrose DGU, this newly developed method exhibits better EVs enrichment effects. The particle concentration and protein content gained by the method 3 are higher than sucrose DGU (approximately 1 × 10^9^ particles/100 mg tissue; 6 μg protein/100 mg tissue) published previously [30]. In term of the particle/ protein ratio, our method ((2.75 ± 0.39) × 10^9^ particles/μg protein) is better than sucrose DGU (approximately 1 × 10^8^ particles/μg protein) described previously [30]. It is worth noting that cedsEVs have a lower purity and smaller in particle diameter compared to tdsEVs. It is possible that cedsEVs contain some small impurities which could not be removed through 0.22-μm filters. Although the Fig. 2a-b showed that the particle numbers and protein content gained by method 1 are the highest among the three methods, its CV% value is large which means poor reproducibility of the data (Fig. 2f).

Our findings suggest that an optimal combination of enzyme concentration and incubation time is a key for effectively releasing sEVs from tissues. For example, a combination of collagenase D of 4 mg/mL, DNase I of 80 U/mL with an incubation time of 20 min gives us the best yield.

In general, sEVs are mostly obtained from fresh or frozen tissues [30, 31]. Recently, a few studies have explored to extract sEVs from cultured tissues slices, which may represent a new source for sampling sEVs [15, 32]. Nevertheless, it remains to be determined whether the physiochemical characteristic of sEVs derived from fresh, frozen tissue or cultured tissue slices are the same. We analyzed the sEVs derived from these specimens. By comparing matched cedsEVs, fresh and frozen tdsEVs, we find that cedsEVs have a smaller size (< 70 nm) (Fig. 4c). TEM also showed that the diameter of cedsEVs is smaller (Fig. 4g). CedsEVs also contain a lower particle number and protein concentration and a smaller ratio of vesicles/particles (Fig. 4a-b and d-f). In addition, the size distribution range of cedsEVs does not fully match the size range of normal sEVs (30–150 nm). Therefore, we speculate that cedsEVs may contain a great number of exomeres (non-vesicular particles <50 nm). It remains possible that our method may not be suitable for the isolation of cedsEVs. An interesting phenomenon was discovered when the surface protein biomarkers of sEVs derived from three types of specimens were examined by NanoFCM and western blotting. NanoFCM analysis showed that the CD9 and CD63 can be detected in all samples and the fluorescence intensity of CD63 in cedsEVs was significantly higher than others (Fig. 5a-b). However, the results of western blotting showed that the Hsp70, CD9 and TSG101 were undetected in the cedsEVs and that CD63 protein was present in all samples (Fig. 5c). We suggested that two potential reasons may contribute to this inconsistence: (1) CD9 protein presents in non-EV materials [22]; (2) TdsEVs and cedsEVs originate from different tumor microenvironments. TdsEVs obtained from the interstitial space of tissue have been secreted in vivo. Whereas cedsEVs are derived from tissue in vitro, these sEVs are of relatively homogenous origin and only reflect the microenvironmental state of tissue block. Coomassie blue staining also revealed that cedsEVs have fewer protein species than others. Additional analyses, such as proteomics or transcriptomics, are needed to identify the different protein species between cedsEVs and tdsEVs.

In conclusion, we developed a convenient method for the isolation and purification of sEVs from liver cancer tissue. Our data suggest that an optimal combination (collagenase D of 4 mg/mL, DNase I of 80 U/mL and incubation time of 20 min) and the interstitial space of tissue are key factors for isolating high quality sEVs from liver cancer tissues. We are currently applying this protocol to different tissues to validate its feasibility and efficiency. Furthermore, we propose that factors such as enzyme concentrations, incubation times and the states of tissues are keys to be considered when developing methods for isolation of sEVs from various tissues.

## Supplementary Information

ESM 1 (DOCX 646 kb)

## Data Availability

All data generated or analyzed during this study are included in the article.
