# Supplementary material for: Comparison of the Variability of Small Extracellular Vesicles Derived from Human Liver Cancer Tissues and Cultured from the Tissue Explants Based on a Simple Enrichment Method
Source: Stem Cell Rev Rep. 2021 Sep 22;18(3):1067–77. doi: 10.1007/s12015-021-10264-1 (PMC8942897; doi:10.1007/s12015-021-10264-1)
Supplement: Supplementary file 1 — (DOCX 646 kb) [file 12015_2021_10264_MOESM1_ESM.docx]

Fig. S1 **NanoFCM analysis of tdsEVs derived from different combinations obtained by three different methods.** (a-c) (i) Representative SSC burst traces of tdsEVs preparation by method 1 (a), method 2 (b) and method 3 (c). (ii) SSC distribution histograms of tdsEVs preparation by method 1 (a), method 2 (b) and method 3 (c). Three methods for isolation sEVs from tissues treated with different concentrations of enzymes (1) (2) (3) and incubation times (20 and 30 min). The samples were treated with PBS and Triton X-100 on ice for 1 h. P is the abbreviation of PBS, for the control group. T is the abbreviation of Triton X-100, for the test group. The particle number measured in one minute was recorded by NanoFCM, as shown in the figure.





Fig. S2 **Expression of CD63 on tdsEVs derived from different combinations obtained by method 3.** (a) Representative SSC burst traces of tdsEVs (i) and PBS (ii) preparation by method 3. (b) SSC distribution histograms of the sEVs (i) and PBS (ii) derived from data collected over 1 min each. The blue rectangular boxes of the SSC histograms mark particles of larger sizes that were presented in the tdsEVs or PBS. (c) Bivariate dot-plots of FITC fluorescence versus SSC for PBS and tdsEVs preparation by method 3. The sEVs were labeled with FITC-conjugated mAbs specific to CD63. The percentages of FITC-positive sEVs are shown in each plot (Left panel); Measured percentages of a specific CD63-positive sEVs (n = 3) of the different combinations were analyzed by unpaired two-tailed Student’s t test and expressed as mean ± SEM. ns: no significant difference (Right panel).


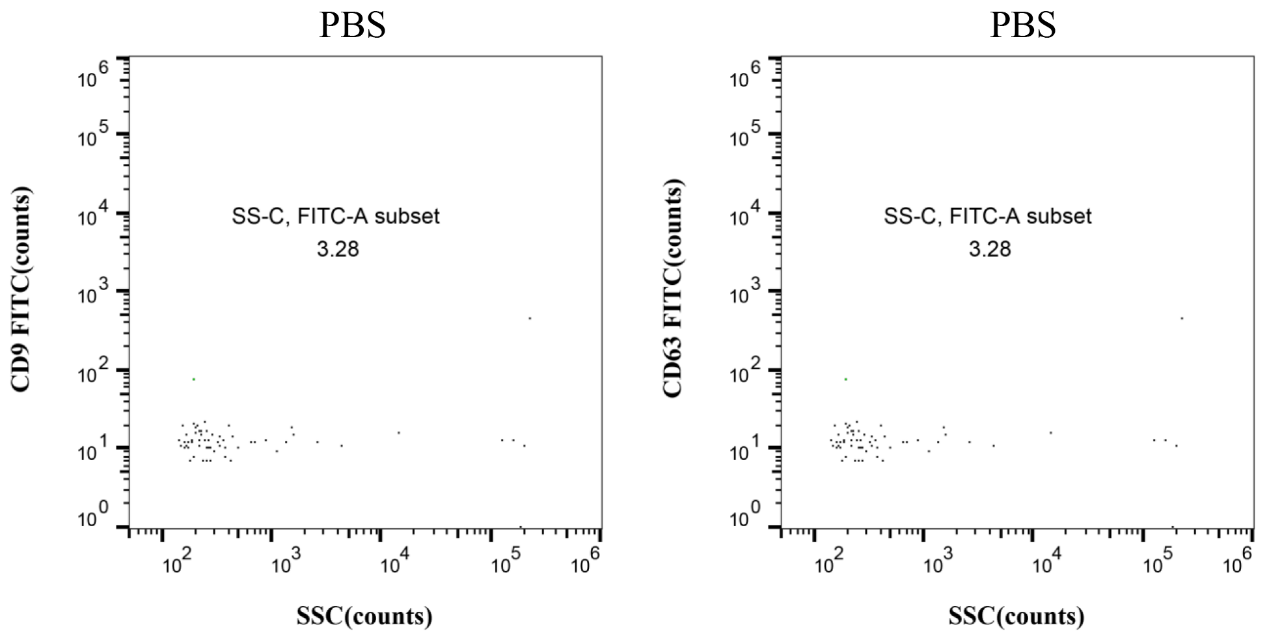


Fig. S3 Bivariate dot-plots of FITC fluorescence versus SSC for PBS as a blank control.
